# Hepatitis A in Puglia (South Italy) after 10 years of universal vaccination: need for strict monitoring and catch-up vaccination

**DOI:** 10.1186/1471-2334-12-271

**Published:** 2012-10-25

**Authors:** Maria Chironna, Rosa Prato, Anna Sallustio, Domenico Martinelli, Silvio Tafuri, Michele Quarto, Cinzia Germinario

**Affiliations:** 1 Department of Biomedical Sciences and Human Oncology - Section of Hygiene, University of Bari, Piazza G. Cesare 11, Bari, 70124, Italy; 2 Department of Medical and Occupational Science - Section of Hygiene, University of Foggia, Foggia, 71100, Italy; 3 Puglia Regional Epidemiological Observatory, Bari, Italy

**Keywords:** Environment, Hepatitis A vaccination coverage, Phylogenetic analysis of HAV, Puglia, Seroepidemiology

## Abstract

**Background:**

Raw seafood consumption was identified as the major risk factor for hepatitis A during the large epidemic of 1996 and 1997 in Puglia (South Italy). In Puglia, vaccination for toddlers and preadolescents has been recommended since 1998.

The aim of the study was to evaluate the incidence, seroprevalence, molecular epidemiology, and environmental circulation of hepatitis A virus (HAV) in Puglia more than ten years after the introduction of anti-HAV vaccination in the regional immunization program.

**Methods:**

Data on the incidence of acute hepatitis A in Puglia were analyzed. Characteristics and risk factors of 97 acute hepatitis A cases occurring in 2008–2009 were analyzed. Serum samples from 868 individuals aged 0 to 40 years were tested for anti-HAV antibodies. Fecal samples from 49 hepatitis A cases were analyzed by sequence analysis in the VP1/P2A region. In 2008, 203 mussel samples and 202 water samples from artesian wells were tested for HAV-RNA.

**Results:**

Between 1998 and 2009, the incidence of acute hepatitis A declined from 14.8 to 0.8 per 100,000. The most frequent risk factors reported by cases in 2008–2009 were shellfish consumption (85%) and travel outside of Puglia or Italy (26%). Seroepidemiologic survey revealed high susceptibility to HAV in children and adults up to age 30 (65%-70%). None of the mussel or water samples were HAV-positive. Phylogenetic analysis revealed co-circulation of subtypes IA (74%) and IB (26%) and clustering of strains with strains from Germany and France, and those previously circulating in Puglia.

**Conclusion:**

Vaccination and improved sanitation reduced the incidence of hepatitis A. Strict monitoring and improved vaccination coverage are needed to prevent disease resurgence.

## Background

In Italy, the epidemiologic pattern of hepatitis A virus (HAV) infection has markedly changed over the past few decades, due to improvements in hygiene and socioeconomic advancements. As a result, Italy has gradually shifted from having a high endemicity status to having a relatively low/intermediate endemicity status [1].

Data from the Integrated Epidemiological System for Acute Viral Hepatitis (SEIEVA) indicate that the incidence rate of acute hepatitis A declined from 4/100,000 in 1991 to 2.2/100,000 in 2009 with a peak during 1996–1998 due to an outbreak in the Puglia region [2]. Analysis of risk factors in the period during 2001–2006 indicated that contact with acute hepatitis A, travel to endemic areas, ingestion of raw shellfish, and cohabitation with day-care age children were the main risk factors [3].

Several serologic studies describe decreased anti-HAV antibody prevalence among individuals under 30 years of age. In particular, a sero-survey conducted among military recruits in 1981, 1990, and 2003 showed a drop in the anti-HAV prevalence from 66% to 29% and to 5%, respectively [4]. The growing number of susceptible young adults consequently increases the likelihood of symptomatic disease following contact with HAV and a greater risk for a severe disease course and complications.

In the Puglia region, located in southeast Italy with a population of approximately 4 million, hepatitis A was endemic between 1989–1995 with an annual incidence ranging from 5 to 70 per 100 000 inhabitants. Incidence rates were typical of endemic areas with a large circulation of HAV. Epidemics were recorded in 1992 and 1994 (involving 2805 and 1349 persons, respectively), with seasonal peaks in February and July–August for both years. An even greater epidemic was reported in 1996 and 1997, with more than 5000 cases per year and incidence rates peaking to 130 cases per 100,000 inhabitants in 1996 [5]. Environmental, food-borne, and behavioral risk factors caused the endemic state of HAV infection in Puglia. In particular, the consumption of raw shellfish was the most relevant exposure source for HAV infection in the endemic and epidemic periods [5-7].

After the large HAV epidemic in 1998 in Puglia, a vaccination program for toddlers and preadolescents was introduced. This vaccine was offered free to all children from 15 to 18 months of age and to preadolescents 12 years of age. Until 2003, a combined hepatitis A plus B vaccine had been used for vaccination of preadolescents as part of the national hepatitis B immunization program. In 2003, this type of vaccination was stopped for 12-year-old preadolescents [8]; only hepatitis A vaccines containing one antigen are now used. No catch-up vaccination campaign has been planned [9].

The aim of the present study was to evaluate the temporal trends of the incidence of acute hepatitis A, the seroprevalence of HAV infection, the molecular epidemiology, and the environmental circulation of the virus in Puglia, more than 10 years after the widespread epidemic of hepatitis A occurred in the years 1996–1997 and following the introduction of anti-HAV vaccination in the regional immunization program.

## Methods

### Routine epidemiologic data

Acute hepatitis A has been a reportable disease in Italy since 1985. The Integrated Epidemiological System for Acute Viral Hepatitis (SEIEVA) is coordinated by the Italian National Institute of Health and involves a network of local health units [2,10]. In the Puglia region, all local health units are involved in this surveillance system and report acute viral hepatitis to SEIEVA, which defines cases based on clinical and serologic criteria and a two-page standard questionnaire for collecting data on risk factors [3]. Data through 2009 were available. The incidence rates for the period during 1998–2009 were calculated using the population of the Puglia region during the same time as the denominator.

### Statistical analysis

Data regarding the characteristics and risk factors for acute hepatitis A cases that occurred in 2008 and 2009 were analyzed. The crude odds ratios (OR) and the 95% confidence intervals (CI) for the risk factors were calculated by univariate analysis. Patients with acute hepatitis B and C reported to SEIEVA in the same period were used as controls. Statistical analysis was performed using EpiInfo, version 6.04d.

### Seroepidemiology

Serum samples from individuals aged 0 to 40 years were collected during 2008. Sera from children up to 15 years of age were obtained using leftover serum from specimens obtained for diagnostic and check-up testing from six regional laboratories (2 located in the Province of Bari, 1 in the Province of Brindisi, 1 in the Province of Foggia, 1 in the Province of Lecce; no laboratory in the Province of Taranto). Serum specimens from subjects older than 15 years of age were randomly selected from the laboratory stock of the Regional Reference Centre for HIV Diagnosis and Prevention of Azienda Ospedaliero-Universitaria Consorziale Policlinico of Bari. Samples from individuals known to be HIV-seropositive were excluded. Samples were collected anonymously, according to the HIV testing policy of the Laboratory. According to the present Italian Data Protection Act, patient initials, sex, year of birth, and date of the sample collection were recorded. Because the study was conducted in accordance with the Data Protection Act, ethical approval was not required for this study. Sera were stored at −20°C until testing.

A total of 868 serum samples were tested for detection of anti-HAV-IgG antibodies. Antibody detection was performed using a commercial test (Architect HAVAb-IgG, Abbott Diagnostics, Rome, Italy) according to the manufacturer’s instructions.

Because it was not possible to assess the vaccination status of the subjects or to discriminate vaccine from natural infection antibody response, age-specific rates of susceptibility to HAV infection were calculated, along with the corresponding 95% confidence intervals. Confidence intervals at 95% (95% CI) were calculated using the modified Wald method. The χ2-test was used to compare categorical variables. A p-value of less than 0.05 was considered statistically significant.

### Molecular epidemiology

Fecal samples were anonymously collected from hepatitis A cases that occurred during 2008 and 2009 and reported to SEIEVA and analyzed by sequence analysis at the VP1-P2A junction. Fecal samples were obtained from all hepatitis A cases reported from a regional health unit to the regional surveillance system as acute viral hepatitis. Samples were collected on a voluntary basis and informed consent was obtained from each patient or parent.

RNA was extracted from 200 μl of fecal extract using a commercial kit (High Pure Viral Nucleic Acid, Roche Diagnostics, Milan, Italy). Elution was performed in a volume of 50 μl. The region at the VP1/2A junction of the HAV genome was amplified. HAV-RNA was amplified by reverse-transcription PCR (RT-PCR), followed in the case of negativity by second-round PCR with the previously reported primers and protocols [11]. PCR products were purified using the QIAquick Purification kit (Qiagen).

For positive samples, nucleotide sequence analysis was performed directly on the purified first or second PCR products using primers for amplification and an ABI PRISM BigDye Terminator Cycle Sequencing Kit (Applied BioSystems, Foster City, CA) in an ABI PRISMA 3130 XL DNA Analyzer (Applied BioSystems).

Phylogenetic analysis was performed using MEGA5 Molecular Evolutionary Genetics Analysis software (http://www.megasoftware.net/mega4/). The genetic distance was calculated using the uncorrected distance algorithm within the distances program in MEGA5. Final tree construction was based on the unweighted pair group method with arithmetic mean (UPGMA) algorithms. The nucleotide sequences of HAV isolates from the patients were compared with those of HAV strains retrieved from the DDBJ/EMBL/GenBank database.

### Environmental and food monitoring

#### Mussels

Extensive case control studies and molecular investigation have confirmed that the consumption of raw seafood is the most relevant exposure source for acquiring HAV infection in the Puglia region [5-7]. In 2008, a total of 203 shellfish samples (Mytilus galloprovincialis) were collected from markets located around the region during an 8-month period (from December to July). Mussels were washed, scrubbed under clean running water, and opened with a sterile knife. The digestive gland (hepatopancreas) was homogenized in a blender for 1 min at maximum speed. Ten grams of homogenate of each sample was then stored at −80°C until testing for the presence of HAV-RNA.

After thawing, homogenate samples were processed and subjected to detection of HAV viral nucleic acid as previously described [7]. We also used nested PCR methods to check for the presence of other enteric viruses (Norovirus, Rotavirus, and Enterovirus).

#### Water

Water samples were collected from 202 artesian wells located all over the region in 2008 and tested for HAV. These artesian wells are continuously monitored for bacterial contamination by the Regional Agency for Environmental Protection because the water may be used in periods of deficiency or in emergency situations.

The water temperature and pH of the samples were determined on site immediately after sample collection. The samples were stored in plastic bottles on ice and delivered to the laboratory within a few hours after collection. All of the samples were assayed for total coliforms according to protocols of the Decreto Legislativo 31/2001 [12].

The occurrence of HAV in water samples was determined using the cation-coated filter method, as previously reported [13,14], followed by nested PCR. In brief, 5 ml of 250 mM AlCl_3_ was passed through an HA filter (0.45μm pore size and 90 mm diameter, Millipore, Milan, Italy) and then 500 ml of the well water sample was passed through the filter. The filter was rinsed with 200 ml of 0.5 mM H_2_SO_4_ (pH 3.0) to remove aluminum ions, followed by elution of viruses with 10 ml of 1.0 mM NaOH (pH 10.8). The filtrate was recovered in a tube containing 50 μl of 100 mM H_2_SO_4_ (pH 1.0) and 100 μl of 100 Tris-EDTA buffer (pH 8.0) for neutralization, followed by centrifugation using the Centriprep YM-50 (Millipore). The Centriprep YM-50 is a centrifugation unit equipped with an ultrafiltration membrane that can achieve the high concentration efficiency needed for viruses. The filtrate was added to the Centriprep YM-50 and centrifuged according to the manufacturer’s protocol. Water samples were processed according to the previous method with modification of the elution volume (500 μl instead of 700 μl). The final concentrated samples were stored at −20°C. Nested PCR methods were used to test for the presence of other enteric viruses (Norovirus, Rotavirus, and Enterovirus).

HAV-RNA was extracted from 200 μl of concentrated samples using a commercial kit (High Pure Viral Nucleic Acid, Roche Diagnostics, Milan, Italy). RNA was eluted in a volume of 50 μl. The nested PCR for HAV-RNA detection was performed using primers in the 5^′^ N-terminal region, as previously described [15].

The detection limit of HAV by nested PCR was determined by seeding 500 ml of well water samples (previously sterilized) with serial 10-fold dilution of an HAV stock solution (1.4 × 10^7^ PFU/ml). The observed detection limit was between 8.2 ×10^-4^ and 8.2 × 10^–2^ PFU per PCR tube.

## Results

### Routine epidemiologic data

**Figure 1:**
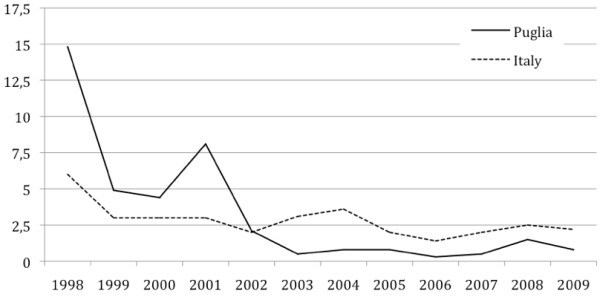
Hepatitis A incidence rates (x 100.000) in Puglia during the years 1998–2009 (SEIEVA).

Between 1998 and 2009, the incidence rates of acute hepatitis A in Puglia declined from 14.8 cases/100,000 to 0.8/100,000 (data from SEIEVA; Figure 1). In 2001, the incidence peaked, reaching 8.1 cases/100,000. Since 2002, the incidence has gradually declined, with the lowest incidence recorded in 2006 (0.3/100,000). Beginning in 2002, the annual incidence rate of hepatitis A in Puglia has remained steadily lower than that reported for Italy.

A detailed analysis of 97 hepatitis A cases that occurred during the years 2008–2009 is shown in Table 1. Males comprised 67% of the hepatitis A cases and females 33%. The majority of cases were adults aged 25 to 34 years of age (44%), 25% were 35 to 44 years of age, and 24% were 15 to 24 years of age. Hospitalization was reported for 93% of cases. Shellfish consumption followed by travel outside the region or outside Italy were the most frequently reported risk factors (85% and 26% respectively) in the 6 weeks before disease onset (Table 2). Four cases (4.1%) were secondary cases. In 2008, a case of fulminant hepatitis requiring liver transplantation was registered. This 24-year-old male reported consumption of raw shellfish as the only risk factor.

**Table 1:** Characteristics of acute hepatitis A cases in Puglia, 2008-2009

**Characteristics**	**No. (%)**
*Sex*	
M	65 (67%)
F	32 (33%)
*Age (years)*	
0-14	3 (3%)
15-24	23 (24%)
25-34	43 (44%)
35-44	24 (25%)
≥45	4 (4%)
*Hospitalization*	
Yes	90 (93%)
No	7 (7%)

**Table 2:** Frequencies and odds ratio of risk factors by acute hepatitis A cases and controls (hepatitis B and C cases occurred in 2008–2009)

**Risk factors**	**Hepatitis A**	**Controls (hepatitis B and C)**	**OR**	**95% CI**
Contact with a jaundice case	1 (1%)	0 (0%)	-	-
Shellfish consumption	83 (85%)	4 (23%)	19.2	4.8-83.2
Raw shellfish consumption	78 (80%)	3 (18.0)	19.1	4.4-94.3
Travel	25 (26%)	2 (12%)	2.6	0.5-17.7
Household of day-care child	12 (12%)	3 (18%)	0.66	0.1-3.3
Intravenous drug use	1 (1%)	0 (0%)	-	-
Well-water drinking	3 (3%)	1 (6%)	0.51	0.0-13.58

### Seroepidemiology

Of the 868 serum samples tested, 502 (57.8%, CI: 54.55-61.12) were negative for anti-HAV IgG antibodies (Table 3). The age classes with the majority of susceptible subjects were children 6 to 10 years of age and young adults, 21 to 25 years of age (70.9, CI: 62.10-79.65 and 70.0%, CI: 61.02-78.98, respectively). A very high prevalence of susceptibility (69.9%) was also detected in children 0 to 5 years of age and in adults 26 to 30 years of age (69.4%). The lowest prevalence of susceptibility was observed in those 16 to 20 years of age (22.1%, CI: 14.47-29.78). The prevalence of susceptibility decreased beginning at age 31 to 35 years, and was 46.5% (CI: 37.90-55.12) in subjects aged 36 to 40 years.

**Table 3:** Prevalence of susceptibility to HAV infection by age class

**Age class (years)**	**Years of birth**	**No. Tested**	**Susceptibility (%)**	**95% CI**
≤5	2008-2003	103	72 (69.9)	61.04-78.76
6-10	2002-1998	103	73 (70.9)	62.10-79.65
11-15	1997-1993	86	56 (65.1)	55.04-75.19
16-20	1992-1988	113	25 (22.1)	14.47-29.78
21-25	1987-1983	100	70 (70.0)	61.02-78.98
26-30	1982-1978	108	75 (69.4)	60.76-78.13
31-35	1977-1973	126	71 (56.3)	47.69-65.01
36-40	1972-1968	129	60 (46.5)	37.90-55.12
**Total**		**868**	**502 (57.8)**	**54.55-61.12**

### Molecular epidemiology

**Figure 2:**
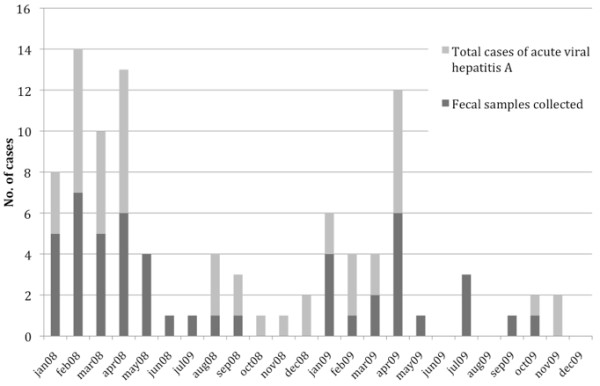
Distribution by month of acute hepatitis A cases during 2008 and 2009.

**Figure 3:**
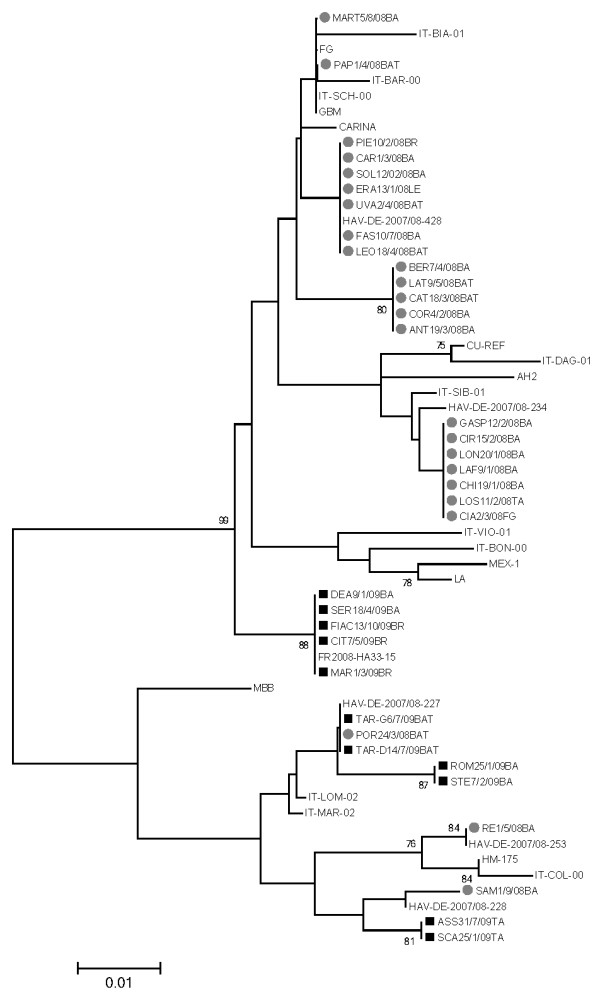
**Neighbor-joining phylogenetic tree of the VP1/2A junction (nt. 3024–3191) showing the relationship between wild-type HAV isolates from this study and other HAV strains.** Gray dots indicate strains of 2008 and black squares indicate strains of 2009. Also the date of clinical onset and the province of provenience [BA (Bari), BR (Brindisi), LE (Lecce), TA (Taranto, BAT (Barletta-Andia-Trani), FG (Foggia)] are indicated. Reference strain sequences for different HAV genotypes were analyzed together with sequences of strains previously characterized in Puglia. Bootstrap probabilities (>70%) are shown at the branches.

A total of 97 acute hepatitis cases were reported to SEIEVA in Puglia in 2008–2009. Fecal samples were obtained from 49 of these cases (29 in 2008 and 23 in 2009; Figure 2). The number of cases peaked between January and April 2008, whereas the number of cases peaked in April in 2009 (12 cases). The Regional Epidemiologic Observatory received no official notice of HAV outbreaks during 2008–2009. HAV-RNA was detected in 35 samples (24 in 2008 and 11 in 2009). The nucleotide sequences of the VP1/2A region of these 35 patients were used for the phylogenetic analysis (Figure 3). The majority of strains isolated in 2008 (87.5%) were genotype IA whereas the majority of strains (54.5%) isolated in 2009 were classified as genotype IB. The majority (74%; 26/35) of nucleotide sequences were closely related to representative HAV genotype IA strains and classified as genotype IA (21 strains of 2008 and 5 strains of 2009), and the remaining 26% (9/35) were classified as genotype IB (3 strains of 2008 and 6 strains of 2009). Strains belonging to subtype IA isolated in 2008 formed three main clusters. Seven identical strains of the first cluster showed 100% identity with the strain HAV-DE-2007/2008/08-428 isolated in Germany in the year 2007. A second distinct cluster of identical strains showed a higher similarity rate (99.3%) with a strain (IT-SCH-00) isolated in Puglia in the year 2000. A third main group of 7 identical strains showed higher similarity (96.8%) with the strain HAV-DE-2007/08-234 and clustered with another “Puglian” strain isolated in 2001 (IT-SIB-01). All IA strains isolated in 2009 were 100% identical and showed 99.4% similarity with a strain isolated in France during an outbreak among homosexual men (FR2008-HA133-15). No linkage emerged among the cases of this cluster. The HAV strain from fulminant hepatitis cases (POR24/3/08BAT) was genotype IB and showed 100% identity with the other two strains characterized in 2009 and also related to HAV IB variant (IT-MAR-02) strains previously reported in Puglia during an outbreak associated with a foodhandler [16]. The other IB strains clustered with different strains previously characterized in Germany during 2007/2008.

### Environmental and food control

#### Mussels

HAV-RNA was not detected in any of the mussel samples. Five samples (2.5%) were positive for Norovirus, 4 samples (2.0%) were positive for Rotavirus, and no samples were positive for Enterovirus.

#### Water

None of the water samples obtained from artesian wells were positive for HAV-RNA. Four samples (2.0%) were positive for Norovirus, 1 sample (0.5%) for Rotavirus, and no samples were positive for Enterovirus.

## Discussion

Hepatitis A has been a serious public health problem in Puglia. The disease has had detrimental effects on the local economy, which is based on tourism and trade of food products, in terms of image and perception of health risk.

Following the large epidemic of 1996–1997, the incidence of hepatitis A in Puglia has steadily declined since 2002. It is conceivable that soon after the large epidemic of 1996–1997 the incidence decreased due to the natural immunization of the population. The incidence rates have remained lower than those in the rest of Italy, reaching a minimum value in 2006. In 2008, however, the number of registered cases began to increase, but in 2009 the incidence rate again declined to 0.8 cases/100,000.

One crucial point for hepatitis A control in Puglia is to determine if the actual policy of universal vaccination of toddlers and adolescents in the region is an effective measure for long-term control of the disease and if the epidemiologic features of HAV infection and the environmental controls are reflected by a real improvement of the situation.

The vaccination coverage levels for hepatitis A in Puglia in 2008 estimated in the national EPI-survey Indagine COnoscitiva di copertura vaccinale NAzionale nei bambini e negli adolescenti (ICONA) was 65% in children 12 to 24 months of age and 68% in adolescents 12 years of age [17]. Routine coverage level data from local health unit vaccination registries are in agreement with the previous figures [18]. The prevalence of susceptibility we found in the present study, however, seem to be very high in all age groups, including those that should have been vaccinated according to the schedule used in the region, and conflicts with the previous estimates. In children and adolescent under 15 years of age, 65% to 70% of subjects do not have detectable anti-HAV antibodies. In addition, older age groups show very high rates of susceptibility to hepatitis A infection, especially adults between 21 to 30 years of age. The age group with the lowest rate of negatives to anti-HAV antibodies comprises those 16 to 20 years of age. The explanation for this observation is the presence of subjects vaccinated with the combined hepatitis A plus B until 2003 as part of the national hepatitis B immunization program. When the hepatitis B vaccination program for adolescents ended, only hepatitis A vaccines containing one antigen were used for immunization and the coverage levels have likely dropped, as shown by the seroprevalence data in younger subjects. This is probably due to the low risk perceived for hepatitis A which is generally considered a mild disease. A coverage rate of less than 20% among children 15 to 18 months of age was previously reported [9], whereas the coverage estimates for the same age groups in 2008 seem overestimated based on the present seroepidemiologic data. It should be noted that serum samples from subjects over 15-year-old tested for HAV IgG were collected at Regional Reference Center for HIV testing. Although this center is the only one in the region that offers a “counselling service” and many subjects come to this center from all around the region, it might be possible that the individuals tested are not representative of the whole population of Puglia. It is conceivable, that the low incidence rate of hepatitis A reported in Puglia is due only partially to the vaccination campaign. The fact that no outbreaks have been reported to date in the region might be attributed to both a persisting “honeymoon” effect and to further improvements in standards of living and hygiene. A recent study indicated that low vaccination coverage levels and improvements in hygienic conditions could be sufficient to control hepatitis A in Puglia [19]. If the vaccination coverage levels do not improve in the future, however, and the rate of susceptibility remain high, then a possible resurgence of HAV cannot be excluded. The trigger could be contaminated food, particularly, raw seafood.

In Puglia the main source of infection is represented by the consumption of contaminated raw mussels [5,6]. HAV contamination of mussels occurs both in the marine environment and in fish market stands where contaminated seawater is often used to wash shellfish, and previous surveillance of shellfish commercialized in the region in the years 1999–2000 revealed the presence of HAV-RNA both in non-depurated and depurated mussels [7]. There is currently no evidence, however, of the presence of HAV in samples collected in 2008. More strict controls on the provenience of such foods and the definitive prohibition of the local custom to store the shellfish in seawater obtained from the urban coast where sewage contamination is likely, may have contributed to the improvement of sanitary conditions for raw seafood. Also, water samples resulted negative for the presence of HAV confirmed a drastic reduction of the circulating virus in the environment, despite the fact that not all urban centers have effective sewage treatment plants [18]. Shellfish consumption, however, remains the main risk factor, as confirmed by the analysis of the risk factors for hepatitis A cases in the years considered in the present study. Continuous monitoring of both shellfish commercialized in the region and risk factors of cases is advisable for the future.

Analysis of the sequences of HAV strains isolated in 2008–2009 showed a co-circulation of IA and IB genotypes. Different clusters were observed both in IA and IB subtypes. The presence of strains identical (from 2008 cases) to a strain previously isolated in Germany in 2007 suggests a probable importation of such HAV strain in Italy. Other IA strains characterized from cases in 2008 were very similar to “Puglian” strains isolated in 2000–2001 and may represent autochthonous strains that have continued to circulate in recent years. IA strains isolated in 2009, in contrast, formed a distinct cluster and seem to have been imported in Puglia. They were highly similar to a strain isolated in France in 2008 during an outbreak in homosexual men, but also very closely related to strains isolated during an outbreak of hepatitis A occurring in Tuscany (Italy) in January-August 2008 [20]. Although there is no evidence for an epidemiologic link between time and localization, this finding suggests successive infections caused by one HAV strain. A probable importation of such strain from Tuscany may be hypothesized. However, the risk of transmission through homosexual activity has not been investigated. Strains belonging to IB subtypes circulating in Puglia in 2008–2009 have high similarity rates with strains isolated in Germany in 2007/2008 and are closely related to an IB variant, IT-LOM-02, previously characterized in the region [16]. The fact that the same nucleotide sequences were detected among patients for two consecutive years also suggests that the same strain was transmitted secondarily.

The incidence of hepatitis A in Puglia has dramatically decreased in the last decade, particularly in very recent years. The current situation is likely due to a combination of different factors such as vaccination and reduced circulation of the virus in the environment due to improved sanitation. The habit of raw seafood consumption and the lack of intervention at urban centers for effective sewage treatment plants persist. Therefore, the still inadequate vaccine coverage levels registered and the high prevalence of susceptibility, as evidenced by the seroepidemiologic survey of the present study, suggest that health authorities should perform strict monitoring because a resurgence of the disease cannot be excluded. A catch-up program to improve vaccination coverage that is targeted especially towards children and young adults is advisable. Continuous health education might also be useful in this context for the effective control of hepatitis A in Puglia.

## Conclusions

The incidence of hepatitis A in Puglia has dramatically decreased in very recent years. The high prevalence of susceptibility suggests that health authorities should perform strict monitoring and a catch-up vaccination program of young adults and children because a resurgence of the disease cannot be excluded.

## Competing interests

The authors declare that they have no competing interest.

## Authors’ contributions

MC and RP participated in the conception of the study, analysis and interpretation of data and drafting the manuscript. AS performed phylogenetic analysis and participated in the interpretation of data. DM, ST and MQ carried out analysis of data and seroepidemiologic study and made a substantial contribution in the acquisition of the data of the study. CG participated in the conception of the study as well as participating in its design and coordination. All authors have read and approved the final manuscript.

## Pre-publication history

The pre-publication history for this paper can be accessed here:

http://www.biomedcentral.com/1471-2334/12/271/prepub
